# Macromolecular Proton Fraction Reveals Divergent White Matter Myelination in Bipolar Disorder and Unipolar Recurrent Depression

**DOI:** 10.3390/bioengineering13010078

**Published:** 2026-01-11

**Authors:** Sofia Gusakova, Liudmila Smirnova, Oleg Borodin, Elena Epimakhova, Alexander Seregin, Vasily Yarnykh

**Affiliations:** 1 Department of Neurology and Neurosurgery, Siberian State Medical University, Tomsk 634050, Russia; borsofya@gmail.com; 2 Mental Health Research Institute, Tomsk National Research Medical Center, Tomsk 634014, Russia; elenaepimakhova@mail.ru (E.E.);; 3 State Regional Autonomous Budget Health Care Institution “Tomsk Regional Oncology Center”, Tomsk 634050, Russia; oyborodin@yandex.ru; 4 Department of Radiology, University of Washington, Seattle, WA 98109, USA; yarnykh@uw.edu

**Keywords:** macromolecular proton fraction, recurrent depressive disorder, bipolar disorder, neuroimaging markers, myelin

## Abstract

Recurrent depressive disorder (RDD) and bipolar disorder (BD) are the most common affective disorders worldwide. However, the pathogenesis of these disorders remains far from understood. Macromolecular proton fraction (MPF) mapping is a sensitive and specific quantitative MRI method for the assessment of brain tissue myelination, but its clinical value for affective disorders remains unknown. This cross-sectional study employed fast MPF mapping on a 1.5 T MRI scanner using the single-point synthetic reference method to investigate myelin abnormalities in white matter of RDD and BD patients. ANOVA revealed a significant main effect of the group (RDD vs. BD vs. two age-matched control groups; F (3.76) = 7.42, *p* < 0.001, η^2^ = 0.227). MPF values were significantly reduced in RDD versus BD patients (*p* < 0.001). BD showed elevated MPF compared to controls (*p* = 0.01). MPF levels showed significant weak-to-moderate correlations with clinical scales of affective disorders. These findings demonstrate divergent cerebral myelination patterns—hypomyelination in RDD versus an increased myelin content in BD. In conclusion, MPF mapping demonstrated a promise as a marker of myelin content changes in affective disorder.

## 1. Introduction

Affective disorders, such as recurrent depressive disorder (RDD) and bipolar disorder (BD), are highly prevalent and functionally debilitating conditions associated with substantial suicide risk and significant societal burden [1]. Despite their high prevalence, a comprehensive pathophysiological understanding of these disorders remains elusive, and validated diagnostic biomarkers are still lacking. Neuroinflammation is commonly viewed as a potential pathological substrate of neurotransmitter dysregulation in affective disorders, particularly in major depressive disorder (MDD) and BD [2,3]. Myelin is a known target of inflammatory response in the central nervous system (CNS) [4]. Postmortem pathological studies reported a decrease in the myelin content [5], reduced expression of myelin-related genes [6], and oligodendroglia apoptosis [7] in white matter (WM) of MDD and BD patients. Recent magnetic resonance imaging (MRI) studies have identified structural brain alterations in patients with depression, including microstructural WM differences suggestive of reduced myelin density [8,9]. Diffusion tensor imaging (DTI) in BD patients has revealed widespread increases in radial diffusivity (RD), which may indicate altered myelination in this disorder [10].

Several other neuroimaging methods including traditional magnetization transfer (MT) imaging [11,12,13,14], inhomogeneous MT imaging [15,16,17], and R1 mapping [18] demonstrated a reduction in quantitative MRI parameters consistent with demyelination in MDD and BD. However, DTI metrics provide only indirect proxies of myelin content [19,20], underscoring the need for more specific and sensitive MRI techniques for the myelin assessment. The lack of specificity to myelin is also a key limitation of traditional MT imaging and R1 mapping [21,22]. A newer approach, inhomogeneous MT imaging [15,16,17], improves specificity to myelin but lacks the capability of absolute quantitation, enabling comparison between different imaging platforms and magnetic field strengths. This technique also has limited availability due to need for a specialized acquisition sequence, which is currently not implemented on most clinical MRI systems.

The fast macromolecular proton fraction (MPF) mapping method [23,24] provides a clinical-ready quantitative myelin imaging approach with a short acquisition time, high reproducibility, and easy implementation based on routine MRI equipment without modification of the original manufacturers’ pulse sequences [24]. MPF represents a key biophysical parameter characterizing the magnetization transfer effect within a two-pool model, quantifying the relative abundance of protons with restricted molecular motion that engage in magnetic cross-relaxation with free water protons. Substantial evidence from animal histologic studies has established MPF as a highly specific myelin biomarker strongly correlated with myelin content in neural tissues [24]. Fast MPF mapping demonstrated sensitivity to myelin content changes in the clinical studies of multiple sclerosis [21], mild traumatic brain injury [25], Parkinson’s disease [26], post-COVID depression [27], and schizophrenia [28]. The last study [28] identified global microscopic myelin deficiency in both white and gray matter in schizophrenia patients, which was significantly associated with the disease duration and negative symptoms. Collectively, the above studies demonstrate high sensitivity of the method for detection of statistically significant intergroup differences within a relatively narrow value range and provide the rationale for applications of the MPF mapping methodology to study myelin abnormalities in affective disorders. The overall objective of this study was to evaluate the feasibility and potential clinical relevance of the fast MPF mapping method in affective disorders. Specifically, our aim was to assess potential changes in global WM myelination in BD and RDD using fast MFP mapping and to examine possible relationships between MPF and commonly used clinical scales characterizing severity of these conditions.

## 2. Materials and Methods

### 2.1. Subjects

A total of 80 participants were examined in this cross-sectional study. The formation of study groups and clinical confirmation of diagnoses of patients with affective disorders was performed by certified physicians of the Affective States Department of Mental Health Research Institute of Tomsk National Research Medical Center during the period from September 2023 to September 2025. Diagnosis was based on both the patient’s subjective complaints and signs of cognitive impairment and affective changes consistent with the pathology. The study included 44 patients, of whom 21 had RDD and 23 had BD. The diagnosis was based on criteria specified in the International Classification of Diseases, 10th Revision (ICD-10). The correctness of the diagnosis was confirmed by scores on the corresponding clinical scales. The examination included a medical history, a physical examination by a general practitioner, and, if necessary, additional laboratory or instrumental studies to rule out active somatic diseases. The criteria for inclusion in the group of patients were the presence of an established diagnosis, age from 18 to 65 years, the absence of acute somatic pathology, the absence of contraindications of MRI, and the capability to sign the informed consent form.

The control group consisted of mentally and somatically healthy individuals without any acute or chronic illnesses and having no contraindications of MRI. The control group was recruited through local advertisements at the Mental Health Research Institute and Siberian State Medical University. Healthy individuals were screened using a self-report questionnaire about lifestyle and health. The questionnaire contains the Buss–Durkee Hostility Inventor (BDHI), Beck Depression Inventory (BDI), and Leonhard’s Personality Inventory (“INDIVIDUAL REGISTRATION CARD”, Supplementary Material). Our questionnaire for healthy individuals was attached after answering the questions. The questionnaire screens for both physical and mental pathology, e.g., endocrine, neurological, and psychiatric disorders. In addition, all individuals included in the control group underwent a complete blood count (CBC) at the Institute’s Clinical Diagnostic Laboratory to rule out acute somatic pathology. The healthy individuals were divided into two subgroups, corresponding to the age ranges of the patient groups. This allowed for a more accurate comparison between the groups of patients and controls with similar age distributions and reduced the influence of age on possible differences in MPF, which is known from the literature [29,30]. The characteristics of the study groups by gender and age are presented in Table 1.

### 2.2. MRI Data Acquisition

All participants underwent non-contrast MRI of the brain using a 1.5 T scanner (Ingenia Evolution, Philips, Amsterdam, The Netherlands). The imaging protocol included both standard clinical sequences and a specialized fast MPF mapping protocol. The MPF mapping protocol was implemented based on a standard manufacturer’s 3D spoiled gradient-echo pulse sequence according to the single-point synthetic reference method [31]. This technique reconstructs MPF maps from three source images with different contrast weightings—magnetization transfer (MT), T1, and proton density (PD)—through voxel-wise iterative fit of the pulsed MT model. All MPF mapping sequences were acquired in the axial plane with in-plane resolution = 1.25 × 1.51 mm, slice thickness = 3 mm (3D field-of-view = 240 × 240 × 240 mm, matrix = 192 × 159 × 80), TE = 4.6 ms, and receiver bandwidth = 179 Hz/pixel. The specific acquisition parameters for each sequence were as follows:

- MT-weighted: TR = 32 ms, flip angle = 12°, acquisition time = 4 min 11 s
- T1-weighted: TR = 20 ms, flip angle = 25°, acquisition time = 2 min 37 s
- PD-weighted: TR = 20 ms, flip angle = 4°, acquisition time = 2 min 37 s

For MT-weighted imaging, a standard manufacturer’s off-resonance Gaussian saturation pulse was applied with the following parameters: frequency offset = 1.1 kHz, effective flip angle = 520°, and pulse duration = 15 ms.

The standard clinical imaging sequences included 3D T1-weighted fast spin-echo (slice thickness = 1.1 mm, TR = 600 ms, TE = 27.7 ms, sagittal orientation); 2D T2-weighted fast spin-echo (slice thickness = 5 mm, TR = 5331 ms, TE = 110 ms, axial orientation); and 3D T2-weighted FLAIR (slice thickness = 1.1 mm, TR = 4800 ms, TE = 314.5 ms, sagittal orientation).

### 2.3. Image Processing and MPF Map Reconstruction

**Figure 1:**
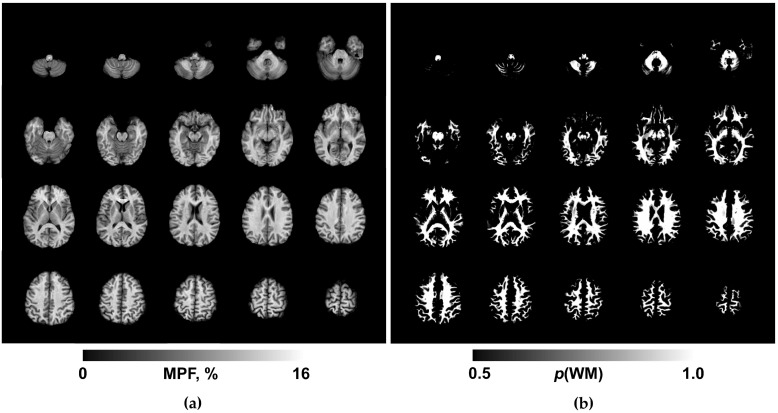
Example 3D MPF (**a**) and WM probability (**b**) maps obtained from a BD patient. Axial cross-sections of a 3D volume are presented with the step of 6 mm (every second cross-section). BD—bipolar disorder; MPF—macromolecular proton fraction; WM—white matter.

Routine clinical images were reviewed by a board-certified radiologist for the presence of focal, diffuse, or structural abnormalities. MPF maps were reconstructed using custom C++ language software (available at https://www.macromolecularmri.org/ (accessed on 15 April 2025)) based on the single-point synthetic reference algorithm [23,31]. The two-pool model parameter constraints were set according to the earlier implementations for 1.5 T [28]: cross-relaxation rate constant R = 19 s^−1^; T_2_ of bound macromolecular protons, T_2_^B^ = 10 μs; and the product of the relaxation rate R_1_ and T_2_ of free water protons, R_1_T_2_^F^ = 0.055. No correction of B_0_ and B_1_ field inhomogeneities was performed, because the effect of these corrections at 1.5 T is negligibly small [32]. An example MPF map obtained using the above acquisition and processing protocol is presented in Figure 1a. The resulting 3D MPF maps were post-processed using FSL software (FMRIB Software Library v. 6.0; Oxford Centre for Functional Magnetic Resonance Imaging of the Brain, University of Oxford, Oxford, UK; available at www.fmrib.ox.ac.uk/fsl/). The post-processing steps included skull stripping using the brain-extraction tool (BET) [33] and tissue segmentation using the fast automated segmentation tool (FAST) [34] under the single-channel three-class approach with the Markov random field parameter 0.1 and four iterations for bias field removal. The resulting WM probability maps (exemplified in Figure 1b) were binarized at the probability threshold of 0.9 to obtain conservative WM masks for MPF quantitation.

### 2.4. Statistical Analysis

All statistical analyses were conducted using Statistica 10 (StatSoft, Tulsa, OK, USA). Normality of residuals in parametric analyses (analysis of variance (ANOVA) or regression) was assessed using the Shapiro–Wilk test. The absence of significant deviations from normal distribution justified the use of parametric methods where appropriate. Clinical ordinal scores were compared between BD and RDD patient groups using the Mann–Whitney U test. Group comparisons of MPF values between patients with BD, RDD, and their respective control groups were performed using one-way analysis of variance (ANOVA) with the participant group as an independent factor. Post hoc pairwise comparisons were conducted using Tukey’s HSD test. Relationships between MPF and clinical variables (age and disease duration) were assessed using Pearson’s correlation coefficient (r). The nonparametric Spearman correlations coefficient (ρ) was used to analyze associations between MPF and ordinal scores on clinical scales. In all analyses, statistical significance was set at *p* < 0.05.

## 3. Results

### 3.1. Psychometric Evaluation

The distribution of clinical presentations in BD patients was as follows: current episode of mixed nature—18 participants; current episode of mild or moderate depression—4 participants; current episode of severe depression without psychotic symptoms—1 participant. Thus, the BD group mainly consisted of patients with a current episode of a mixed nature. In the RDD group, patients were distributed as follows: remission—1 patient; current episode of moderate severity—19 patients; current episode of severe depression without psychotic symptoms—1 patient. Thus, in the RDD, patients with a moderate current episode of depression predominated. The clinical study was conducted using a set of standardized scales and questionnaires before the start of therapy upon admission to the hospital. To assess the severity of current depression, our study used the 24-item version of the SIGH-SAD scale, which includes 17 items from the Hamilton Depression Rating Scale (Structured Interview Guide for the Hamilton Depression Rating Scale, Seasonal Affective Disorders) [35]. A score for typical depressive symptoms, a score for atypical depressive symptoms, and a total score were calculated. Severity was assessed using the Clinical Global Impression Scale (CGI) subscale, CGI-S (Clinical Global Impression—Severity). The Bipolar Spectrum Diagnostic Scale (BSDS) was used to screen patients with bipolar disorder. According to the BSDS, all our patients had a high-to-moderate probability of bipolar disorder. All psychometric assessments were conducted upon admission to the clinic, before treatment was prescribed.

The severity of depressive symptoms is assessed quantitatively using the SIGH-SAD scale, which is calculated using the sum of scores for 17 typical and 7 atypical symptom items. These scores are categorized as mild (scores from 8 to 16), moderate (scores from 17 to 23), and severe (scores equal to or greater than 24).

In the BD group, moderate (33%) and mild (33%) depressive symptoms were predominant. In total, 19% of patients scored less than eight points and thus did not exhibit depressive symptoms. The proportion of individuals with severe depressive symptoms was 15%.

In the RDD group, half of the patients (50%) demonstrated moderate depressive symptoms, while the proportion of patients with severe depression was 45%, and only 5% exhibited mild depressive symptoms.

According to the atypical symptom scale, the severity of symptoms in both groups did not show significant differences (Table 2). However, a characteristic feature of our RDD group was that 35% of patients showed relatively high scores on atypical symptoms.

The severity of the patients’ condition was assessed using the “Severity” subscale of the Clinical Global Impression (CGI), which allows us to assess the severity of the disease before the start of therapy. The median scores on this scale in the study groups are presented in Table 2 and indicate that the condition of patients in both groups is characterized predominantly as “Moderate Disorder” and does not demonstrate reliable differences (*p* = 0.05).

The distribution of BD patients by S-CGI score was as follows: 5%—mild disorder; 43%—moderate disorder; 47%—severe disorder; 5%—very severe disorder. For patients with RDD, the following distribution was observed: 10%—mild disorder; 70%—moderate disorder; 5%—severe disorder; 10%—very severe disorder.

A quantitative assessment of the presence of BD symptoms was conducted using the BSDS. It was shown that upon admission to the hospital, BD patients demonstrated the highest probability of having this disorder (90%) (10% had a moderate probability of BD) and significantly differed in this indicator from patients with RDD (*p* = 0.000) (Table 2). In the RDD group, 15% of patients had a high probability of BD, 10% had a moderate probability, and the vast majority (75%) showed a low probability of BD.

Group comparisons (Table 2) demonstrated that before the start of therapy, the severity of depressive symptoms determined by the total score on the SIGH-SAD scale was significantly higher in patients with RDD (*p* = 0.003). Furthermore, a significant difference was observed between the groups in the severity of typical depressive symptoms (*p* = 0.002), while the severity of atypical symptoms did not differ significantly between the compared groups (*p* = 0.053). Thus, Table 2 clearly demonstrates the accuracy of the clinically established diagnoses of patients in the study groups.

### 3.2. MRI and MPF Analysis

An example of the maps used in the work is shown in Figure 1.

**Figure 2:**
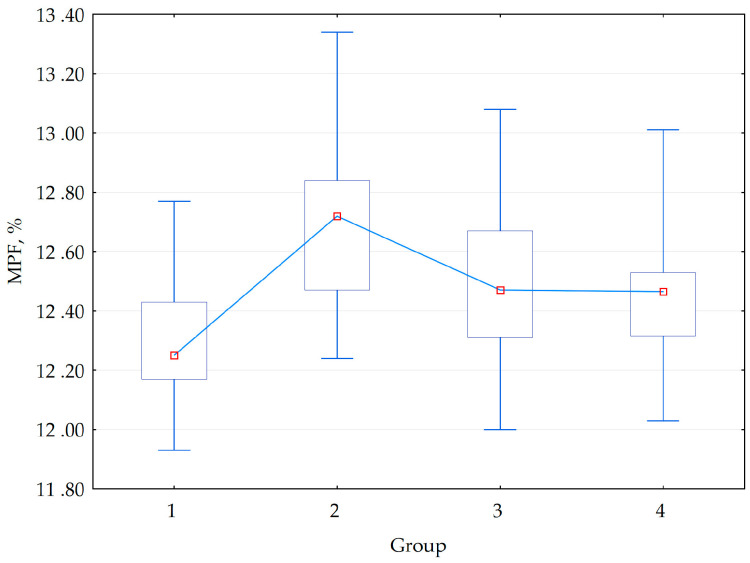
Box-and-whisker diagram representing MPF (macromolecular proton fraction) value distributions in patients’ groups (RDD—1, BD—2, and control group for RDD—Group 3 and BD—4). BD—bipolar disorder; RDD—recurrent depressive disorder. Red circles are median mean for each group, the upper and lower edges of the box means upper and lower quartiles, the ends of whiskers are minimum and maximum observed values.

Clinical MRI results in all participants were radiologically unremarkable. The one-way ANOVA results for WM MPF values are presented in Table 3, and visual representation of group-wise MPF distributions is provided in Figure 2. ANOVA revealed a statistically significant main effect of group membership on WM MPF. The effect size was large (η^2^ = 0.227), indicating that approximately 23% of the variance in MPF was explained by the group differences. The observed statistical power was 0.98, demonstrating a 98% probability that the analysis correctly identified existing intergroup differences and confirming adequate sample size for this study design.

The complete ANOVA results are presented in Table 3, with visual representation of group-wise MPF distributions provided in Figure 2.

Post hoc analysis using Tukey’s HSD test identified statistically significant pairwise differences between the RDD and BD groups, and between the BD patient group and their corresponding healthy controls (*p* < 0.05). Conversely, no statistically significant differences were found between the two control groups (for RDD and BD), despite initial significant age differences during participant recruitment. Detailed results of the post hoc comparisons are summarized in Table 4.

The Pearson correlation analysis demonstrated no significant correlation between white matter MPF and age (r = −0.15, *p* > 0.05), and no correlation was observed between white matter MPF and illness duration (r = −0.20, *p* > 0.05).

In addition, a correlation analysis of the MPF values with the numerical indicators of the clinical scales was performed in the entire sample of examined patients without separation into groups. In our study, the SIGH-SAD and CGI clinical scales were used to assess the severity of depressive symptoms and illness severity, and the BSDS was used to confirm the presence of bipolar spectrum disorders. These measures are qualitative traits, quantified through ranking. Under these conditions, the Spearman correlation coefficient becomes more appropriate, since, unlike the Pearson correlation coefficient, the Spearman correlation coefficient allows for the analysis of relationships between values represented by rank values or scores.

**Figure 3:**
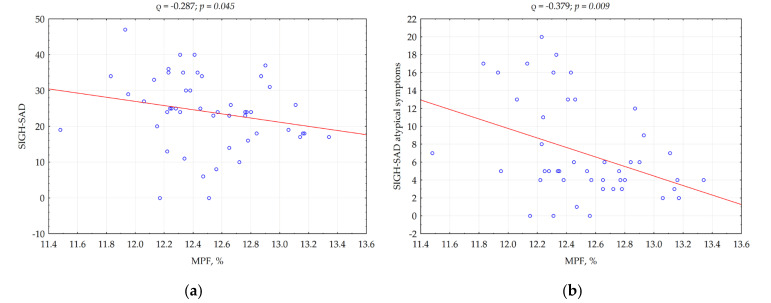

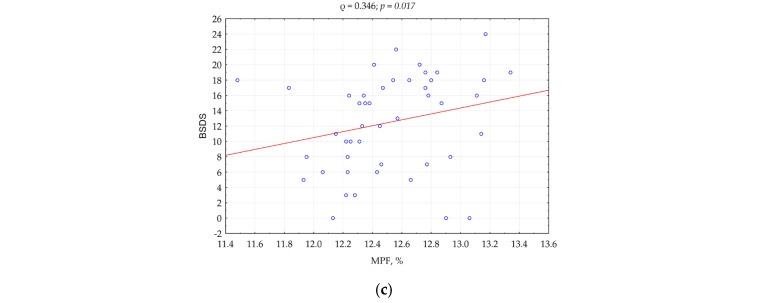
Correlations between MPF (macromolecular proton fraction) and the score on the scale: (**a**)—SIGH-SAD (Structured Interview Guide for the Hamilton Depression Rating Scale, Seasonal Affective Disorders); (**b**)—SAD (Seasonal Affective Disorders) for atypical symptoms; (**c**)—BSDS (The Bipolar Spectrum Diagnostic Scale). Red lines represent the linear regression plots and blue circles correspond to the individual measurements.

Significant negative correlations were identified between WM MPF and the SIGH-SAD scale for both atypical (ρ = −0.379; *p* = 0.009) and general symptoms (ρ = −0.287; *p* = 0.045) (Figure 3a,b). MPF also positively correlated with BSDS (ρ = 0.346; *p* = 0.017) (Figure 3c).

## 4. Discussion

This study presents the first application of the fast MPF mapping method for the quantitative assessment of myelination in patients with RDD and BD. For the first time, we compared myelination changes in patients with RDD and BD not only with healthy individuals, but also between groups of affective disorders. Furthermore, to the best of our knowledge, this is the first report of significant correlations between MPF and clinical scales in affective disorders. A significant reduction in WM myelination in RDD patients is consistent with previous findings in depressive disorders including MDD [5,8,9,11,12,16,17,18] and post-COVID depression [27]. It is important to note that only the last study [27] relied on MPF as a myelin marker and reported a global decrease in MPF across all WM [27]. However, we would like to emphasize that this study presents data only on post-COVID depression, which clearly has a completely different etiology (acute neuroinflammation and a pathological autoimmune response) than RDD. This is the first time that the MPF index has been used to assess myelination changes in RDD. Investigations based on other methods are in line with these findings. Particularly, DTI similarly showed widespread fractional anisotropy (FA) reductions in WM of depression patients with childhood trauma anamnesis, confirming global alterations in WM connectivity [36]. A comparable FA study in MDD patients identified microstructural impairments in three of seven WM tracts: reduced FA in the genu of the corpus callosum showing negative correlation with depression severity; and reduced FA with axial diffusivity (AD) alterations in both superior and inferior longitudinal fasciculi [37]. Furthermore, comparative analysis between patients experiencing current depressive episodes and those in remission revealed more extensive WM abnormalities during acute phases, involving the left insula, left middle occipital gyrus, right thalamus, left pallidum, and left precuneus. During remission, only the left insula maintained significant FA reduction, suggesting global WM impairment specifically during active depressive states [38]. Notably, a comprehensive meta-analysis of DTI data from 5171 subjects demonstrated significant reductions in global WM integrity as measured by FA [39]. Also in agreement with our observations, the findings consistent with myelin loss in depression were reported in earlier magnetization transfer [11,12,13,14,15,16,17] and R1 mapping studies [18].

Correlation analysis in our current study revealed no significant linear association between myelination measures and disease duration, which is known from the literature [29,30], also contrasting with an earlier prospective case–control cohort study utilizing DWI that identified a significant diagnosis time interaction, indicating accelerated decline in superior longitudinal fasciculus (SLF) integrity among MDD patients compared to healthy controls [40]. This discrepancy underscores the necessity for expanded sample sizes in future studies to enhance analytical precision. Our comparative analysis of cerebral WM MPF values revealed substantial differences between BD and RDD groups despite clinical similarities in disease onset that often contribute to BD underdiagnosis. These findings align well with earlier DTI investigations of microstructural WM differences between unipolar depression (n = 562) and depressed patients later converting to BD (n = 83) [41].

Our unexpected finding is an elevated cerebral WM MPF in BD patients compared to both matched controls and RDD patients. This appears to contradict an earlier neuroimaging studies reporting a decrease in MRI parameters associated with myelin in both patient groups [8,9,10,11,12,13,14,15,16,17,18,19,20,21,22,23,24,25,26,28,42,43]. The existing literature presents conflicting evidence, reflecting the pathophysiological complexity of BD and its inherent clinical heterogeneity. Supporting our observations, a resting-state fMRI study of BD patients during depressive episodes revealed enhanced functional connectivity between the superficial temporofrontal network and both cerebellar and pre/post-central networks, potentially indicating functional tract consolidation in these regions. Conversely, a whole-brain voxel-based multicomponent diffusion MRI study employing multiple diffusion metrics identified lower FA in BD patients, but did not find differences in MPF [44]. As such, neuroimaging findings in BD remain inconsistent across studies. A multicenter investigation of neurodevelopmental BD subtypes based on cortical folding patterns reported elevated local sulcal indices (l-SIs) in the right prefrontal dorsolateral region in early-onset BD, but reduced l-SI in the left superior parietal cortex in psychotic BD, though no significant differences emerged between healthy controls and the entire patient cohort [45]. Another possible explanation of an increased MPF in BD may be related to a relatively young age of our BD group and a difference in the brain maturation rates between control subjects and BD patients. This hypothesis is supported by a recent DTI study [46], which reported an earlier peak of brain maturation in BD (27–29 years) as compared to healthy controls (32–36 years). If a similar trend is valid for MPF, it may explain greater MPF values in BD relative to age-matched controls given the fact that the mean age of our groups is 24–25 years. Of note, this study [46] also did not identify differences between BP and controls in any of the DTI metrics. Consequently, given a limited amount of MPF data in BD patients, contradictory DTI and functional MRI findings, and heterogeneous literature reports, we assert that pathogenetic mechanisms underlying myelination abnormalities remain inadequately characterized in BD compared to RDD. This area warrants more extensive investigation with careful consideration of confounding factors including medication effects and disease subtypes, which have been previously established as significant sources of variability [47].

## 5. Limitations

This study has several limitations. First, this was a cross-sectional study, and therefore a mechanistic interpretation of associations between myelin abnormalities and affective disorders would not be possible. Second, we were able to recruit a limited number of patients during the study period, and this circumstance precluded a more detailed stratification of participants across the spectrum and severity of symptoms. Third, the BD and RDD groups differed in age. Therefore, we recruited age-matched healthy controls and tested the age-related relationship of the studied parameter. Fourth, our study did not examine the effect of therapy on changes in white matter myelination. While it cannot be completely excluded, such an effect is rather unlikely. Patients were referred for MRI during the first week of admission, and most of them either were treatment-naive or had not received antidepressant or antipsychotic medications for several years. Fifth, we focused on the global WM analysis and did not assess anatomic sub-regions within WM due limited spatial resolution (slice thickness of 3 mm). Precise WM segmentation would require isotropic high-resolution MPF maps [48], which were unavailable with our MRI equipment.

## 6. Conclusions

This study establishes distinct white matter myelination patterns in affective disorders, demonstrating a reduced myelin content in RDD but paradoxically elevated myelination in BD. These differential myelination profiles may suggest unique neurobiological mechanisms underlying these conditions. The findings of this study position MPF as a promising biomarker capable of detecting subtle changes in myelination and underscore the necessity for multimodal approaches in future neuroimaging studies of mood disorders.

## Figures and Tables

**Table 1:** Characteristics of the study groups by gender and age.

Group	Male				Age Me [Q25; Q75]
	Men		Women		
	%	Abs.	%	Abs.	
Recurrent depressive disorder (n = 21)	19	4	81	17	38 [25; 48]
Bipolar disorder (n = 23)	26	6	74	17	24 [20; 31]
Healthy control subjects for RDD (n = 19)	21	4	79	15	38 [32; 45]
Healthy control subjects for BD (n = 17)	29	5	71	12	25 [23; 32]

Appendix: Abs.—Absolute number of individuals; Me [Q25; Q75]—Median and quartiles; BD—bipolar disorder; RDD—recurrent depressive disorder.

**Table 2:** Differences in scores on clinical scales in groups of patients with bipolar disorder and recurrent depressive disorder.

Scale	Points, Me [Q25; Q75]		*p* (Mann–Whitney U Test)
	BD	RDD	
SIGH-SAD for typical depressive symptoms	15.0 [12.5; 19.0]	20.0 [19.0; 24.0]	0.002
SIGH-SAD for atypical depressive symptoms	4.0 [3.0; 5.0]	6.0 [4.0; 13.0]	0.053
Total score SIGH-SAD	18.0 [14.0; 24.0]	26.0 [24.0; 35.0]	0.003
S-CGI	5.0 [4.0; 5.0]	4.0 [4.0; 4.0]	0.050
BSDS	17.5 [16.0; 19.0]	7.0 [5.0; 10.0]	0.000

Appendix: Me [Q25; Q75]—Median and quartiles; SIGH-SAD—the Hamilton Depression Rating Scale (Structured Interview Guide for the Hamilton Depression Rating Scale, Seasonal Affective Disorders); S-CGI—Clinical Global Impression—Severity; BSDS—The Bipolar Spectrum Diagnostic Scale; BD—bipolar disorder; RDD—recurrent depressive disorder.

**Table 3:** ANOVA analysis results with participant group as the independent factor (RDD—Group 1, BD—Group 2 and their respective healthy control groups for RDD—Group 3 and BD—Group 4).

Source of Variation	Sum of Squares (SS)	Degrees of Freedom (DF)	Mean Square (MS)	F-Statistic	*p*-Value	η^2^	Observed Power (alpha = 0.05)
Group	1.709	3	0.57	7.419	<0.001	0.227	0.981
Within-group (Error)	5.835	76	0.077				
Total	7.544	79					
Intercept	12292	1	12292	160098	<0.001	0.9995	1.000

**Table 4:** Group sample sizes and results of pairwise comparisons using Tukey’s HSD post hoc test.

Group Number	Group	N	Mean MPF ± SD, %	Statistically Significant Differences (*p* < 0.05)
1	RDD	21	12.30 ± 0.22	BD (2)
2	BD	23	12.69 ± 0.33	RDD (1), control group for BD (4)
3	Control for RDD	19	12.48 ± 0.3	Not found
4	Control for BD	17	12.44 ± 0.25	BD (2)

Appendix: BD—bipolar disorder; RDD—recurrent depressive disorder; MPF—macromolecular proton fraction; SD—standard deviation.

## Data Availability

The data presented in this study are available on request from the corresponding author.

## Supplementary Materials

The following supporting information can be downloaded at: https://www.mdpi.com/article/10.3390/bioengineering13010078/s1, Individual Registration Card.

## Abbreviations

The following abbreviations are used in this manuscript:

AD	Axial diffusivity
ANOVA	One-way analysis of variance
BD	Bipolar disorder
BSDS	Bipolar Spectrum Diagnostic Scale
CGI	Clinical Global Impression Scale
CGI-S	Clinical Global Impression-Severity
CNS	The central nervous system
DTI	Diffusion tensor imaging
FA	Fractional anisotropy
GenuCC	Genu of the corpus callosum
ICD-10	International Statistical Classification of Diseases
l-SIs	Local sulcal indices
MDD	Major depressive disorder
MPF	Macromolecular proton fraction
MRI	Magnetic resonance imaging
MT	Magnetization transfer
PD	Proton density
R1	Longitudinal relaxation rate
RD	Radial diffusivity
RDD	Recurrent depressive disorder
SIGH-SAD	Structured Interview Guide for the Hamilton Depression Rating Scale, Seasonal Affective Disorder Version
SLF	Superior longitudinal fasciculus
T1	Longitudinal relaxation time
T2	Transverse relaxation time
TE	Echo time
TR	Time of Repetition
Tukey’s HSD test	Tukey’s HSD Honest Significant Difference test
WM	Cerebral white matter
