# Supplementary material for: Macromolecular Proton Fraction Reveals Divergent White Matter Myelination in Bipolar Disorder and Unipolar Recurrent Depression
Source: Bioengineering (Basel). 2026 Jan 11;13(1):78. doi: 10.3390/bioengineering13010078 (PMC12837752; doi:10.3390/bioengineering13010078)
Supplement: Supplementary file 1 [file bioengineering-13-00078-s001.zip › bioengineering-4057204-supplementary.pdf]

INDIVIDUAL REGISTRATION CARD

№_____________________________

I. Sociodemographic map and questionnaire on the history of somatic pathology

1.Full name:____

2. Year of birth _____________ 3. Age ______________

4. Gender: M/F

5. Height (cm)___________ 6. Weight (kg)____________

7. Waist circumference (cm)__________ 8. Blood Pressure: _____________________

9. Nationality:____

10. Place of birth (region, locality): ____

11. Education: ____

12. Place of residence (region, locality): ______________________________________

13. Place of work (study): ____

14. Profession: ____

15. Marital status:____

16.Comorbid somatic disorders: No/Yes, specify diagnoses: ____

17.Previous psychiatric, neurological illnesses, injuries, concussions: ____

18.Infections contracted in the last 3 months: ____

19. Presence of psychiatric, neurological, or hereditary diseases among close relatives: No/Yes, specify diagnoses and degree of kinship: ___________________________

20.Additional information you consider necessary to mention: _____________________________________

21. Smoking: No/Yes, number of cigarette packs per day: ______

II. Brief Form of Voluntary Informed Consent for Participation in Research

I, give my consent to participate in a scientific research project within the framework of the RSF Project No. 23-75-00023 "Searching for new molecular mechanisms of affective disorders aimed at developing diagnostic and prognostic methods using proteomic approaches and neuroimaging" under the guidance of the PhD MD Lyudmila P. Smirnova, conducted by the research staff of Department of Affective Disordersand the Laboratory of Molecular Genetics and Biochemistry of the Mental Health Research Institute of the Tomsk National Research Medical Center.

Research Location: Mental Health Research Institute, Tomsk National Research Medical Center, Tomsk; 4 Aleutskaya Street.

Contact Information for Researchers: 8 (3822) 72-38-29

I was informed about:

• The scientific nature of the research,

• The objectives of this research,

• The potential benefits and risks, as well as possible discomfort, that participation in the research may entail;

• The potential risks associated with MRI (magnetic resonance imaging) examination; Contraindications for MRI examination (presence of a pacemaker, ferromagnetic or electronic implants in the middle or inner ear, insulin pumps, neurostimulators, large magnetic metallic implants especially in the lungs, metal fragments in the eye, aneurysm clips. The obligation to inform the doctor about the presence of foreign objects (dental crowns, piercings, metal threads, etc.).

• In published works (articles, monographs), my personal data will not be disclosed in illustrated materials.

I have received the above-mentioned information and answers to all my questions in writing.

I consent to having my medical records reviewed by the researcher, with the condition that they maintain professional confidentiality.

I have received assurances that I have the right to discontinue participation in the research and receive the appropriate examination and treatment if needed.

I consent to my data being entered into a computer file, ensuring protection in accordance with the law.

I have the right to access and, if necessary, correct this data through the healthcare provider of my choice.

Participant's Full Name:

Signature:
Date of Consent:

Questionnaire №1

Instructions: The test consists of a series of statements concerning certain life situations. You need to answer "yes" or "no" to each question. Try to answer truthfully and sincerely.

1. Sometimes I can't control my desire to harm others.
2. I talk about people I don't like.
3. I get annoyed easily, but I calm down quickly.
4. If I am not asked nicely, I do not fulfill requests.
5. I don't always get what I deserve.
6. I know that people talk about me behind my back.
7. If I disapprove of my friends, I make them feel it.
8. When I have deceived someone, I experience guilty pangs.
9. I believe I am incapable of hitting another person.
10. I never get so irritated that I throw things.
11. I am always tolerant of others' flaws.
12. If I don't like a rule, I want to break it.
13. Others can almost always take advantage of favorable circumstances.
14. I am cautious around people who are somewhat more friendly than I expected.
15. I often disagree with people.
16. Sometimes I have thoughts I am ashamed of.
17. If someone hits me first, I won't answer back.
18. When I get angry, I slam doors.
19. I am much more irritable than I seem.
20. If someone pretends to be a boss, I always oppose him.
21. My fate slightly troubles me.
22. I think many people don't like me.
23. I can't resist arguing if people disagree with me.
24. People who avoid work should feel guilty.
25. Anyone who insults me or my family invites a fight.
26. I am incapable of making rude jokes.
27. I become furious when I am mocked.
28. When people act like bosses, I do everything to keep them humble.
29. I see someone I dislike almost every week.
30. Quite a few people envy me.
31. I demand that people respect me.
32. I feel guilty that I do little for my parents.
33. People who constantly bother you deserve to be "clipped on the nose."
34. I am never gloomy out of anger.
35. If I am treated worse than I deserve, I don't get upset.
36. If someone gets on my nerves, I ignore it.
37. Although I don't show it, sometimes I feel envious.
38. Sometimes I think people are making fun of me.
39. Even if I am angry, I don't resort to strong language.
40. I want my sins to be forgiven.
41. I rarely retaliate, even if someone hits me.
42. When things don't go my way, I sometimes take offense.
43. Sometimes people irritate me just by being there.
44. There are no people I truly hate.
45. My principle: "Never trust strangers."
46. If someone irritates me, I am ready to say everything I think about him.
47. I do many things I later regret.
48. If I get angry, I might hit someone.
49. Since childhood, I have never shown fits of anger.
50. I often feel like a powder keg ready to explode.
51. If everyone knew what I feel, I would be considered difficult to get along with.
52. I always think about what secret reasons make people do something nice for me.

53. When someone yells at me, I start yelling back.
54. Failures upset me.
55. I fight as often and as infrequently as others.
56. I don't remember times when I was so angry that I grabbed and broke something.
57. Sometimes I feel I am the first to start a fight.
58. Sometimes I feel life treats me unfairly.
59. I used to believe most people told the truth, but now I don't.
60. I only swear out of anger.
61. When I do something wrong, my conscience troubles me.
62. If I need to use physical force to defend my rights, I use it.
63. Sometimes I show my anger by pounding on the table with my fist.
64. I can be rude to people I don't like.
65. I have no enemies who want to harm me.
66. I don't know how to put someone in his place, even if he deserves it.
67. I often think I have lived the wrong way.
68. I know people capable of provoking me to a fight.
69. I don't get upset over small things.
70. Rarely do I think that people are trying to upset or insult me.
71. I often only threaten people, even though I don't intend to follow through.
72. Recently, I have become a bore.
73. I often raise my voice during arguments.
74. I usually try to hide my bad attitude towards people.
75. I would rather agree with something than argue.

QUESTIONNAIRE №2

Instructions: "In each block of four statements, select the one that most accurately describes how you are feeling at the moment."

1.
0 I do not feel sad.
1 I feel sad
2 I am sad all the time and I can't snap out of it.
3 I am so sad and unhappy that I can't stand it.
2.
0 I am not particularly discouraged about the future.
1 I feel discouraged about the future.
2 I feel I have nothing to look forward to.
3 I feel the future is hopeless and that things cannot improve.
3.
0 I do not feel like a failure.
1 I feel I have failed more than the average person.
2 As I look back on my life, all I can see is a lot of failures.
3 I feel I am a complete failure as a person.
4.
0 I get as much satisfaction out of things as I used to.
1 I don't enjoy things the way I used to.
2 I don't get real satisfaction out of anything anymore.
3 I am dissatisfied or bored with everything.
5.
0 I don't feel particularly guilty
1 I feel guilty a good part of the time.
2 I feel quite guilty most of the time.
3 I feel guilty all of the time.
6.
0 I don't feel I am being punished.
1 I feel I may be punished.
2 I expect to be punished.
3 I feel I am being punished.
7.
0 I don't feel disappointed in myself.

1 I am disappointed in myself.
2 I am disgusted with myself.
3 I hate myself.

8.

0 I don't feel I am any worse than anybody else.
1 I am critical of myself for my weaknesses or mistakes.
2 I blame myself all the time for my faults.
3 I blame myself for everything bad that happens.

9.

0 I don't have any thoughts of killing myself.
1 I have thoughts of killing myself, but I would not carry them out.
2 I would like to kill myself.
3 I would kill myself if I had the chance.

10.

0 I don't cry any more than usual.
1 I cry more now than I used to.
2 I cry all the time now.
3 I used to be able to cry, but now I can't cry even though I want to.

11.

0 I am no more irritated by things than I ever was.
1 I am slightly more irritated now than usual.
2 I am quite annoyed or irritated a good deal of the time.
3 I feel irritated all the time.

12.

0 I have not lost interest in other people.
1 I am less interested in other people than I used to be.
2 I have lost most of my interest in other people.
3 I have lost all of my interest in other people.

13.

0 I make decisions about as well as I ever could.
1 I put off making decisions more than I used to.
2 I have greater difficulty in making decisions more than I used to.
3 I can't make decisions at all anymore.

14.

0 I don't feel that I look any worse than I used to.
1 I am worried that I am looking old or unattractive.
2 I feel there are permanent changes in my appearance that make me look unattractive
3 I believe that I look ugly.

15.

0 I can work about as well as before.
1 It takes an extra effort to get started at doing something.
2 I have to push myself very hard to do anything.
3 I can't do any work at all.

16.

0 I can sleep as well as usual.
1 I don't sleep as well as I used to.
2 I wake up 1-2 hours earlier than usual and find it hard to get back to sleep.
3 I wake up several hours earlier than I used to and cannot get back to sleep.

17.

0 I don't get more tired than usual.
1 I get tired more easily than I used to.
2 I get tired from doing almost anything.
3 I am too tired to do anything.

18.

0 My appetite is no worse than usual.
1 My appetite is not as good as it used to be.
2 My appetite is much worse now.
3 I have no appetite at all anymore.

19.

0 I haven't lost much weight, if any, lately.
1 I have lost more than five pounds.
2 I have lost more than ten pounds.
3 I have lost more than fifteen pounds.

20.
0 I am no more worried about my health than usual.
1 I am worried about physical problems like aches, pains, upset stomach, or constipation.
2 I am very worried about physical problems and it's hard to think of much else.
3 I am so worried about my physical problems that I cannot think of anything else.

21.
0 I have not noticed any recent change in my interest in sex.
1 I am less interested in sex than I used to be.
2 I have almost no interest in sex.
3 I have lost interest in sex completely.

QUESTIONNAIRE №3

You are presented with statements concerning your character. Answer spontaneously, without much thought—you need to choose one of two responses: "yes" or "no"—for each question, and there are no other options.

1. Do you often have a cheerful and carefree mood?
2. Are you sensitive to insults?
3. Do tears sometimes come to your eyes when watching movies, at the theater, or during conversations?
4. When you do something, do you doubt whether everything was done correctly and only feel at ease after double-checking?
5. Were you as brave in childhood as your peers?
6. Do your moods often swing sharply from extreme happiness to aversion to life and yourself?
7. Are you usually the center of attention in a social group?
8. Do you sometimes feel irritable without reason, to the point where it's better not to talk to you?
9. Are you a serious person?
10. Are you able to experience enthusiasm or admiration for something?
11. Are you enterprising?
12. Do you quickly forget if someone insults you?
13. Are you tenderhearted?
14. When you drop a letter into a mailbox, do you check by running your hand along the slot to see if it has fully fallen inside?
15. Do you always strive to be among the best workers?
16. Were you afraid of thunderstorms or meeting unfamiliar dogs in childhood (or maybe such feelings still occur now in adulthood)?
17. Do you always try to maintain order in everything and everywhere?
18. Does your mood depend on external circumstances?
19. Are your acquaintances fond of you?
20. Do you often experience inner anxiety or a sense that something bad or unpleasant may happen?
21. Do you often have a somewhat depressed mood?
22. Have you ever had a temper tantrum or nervous breakdown at least once?
23. Is it difficult for you to sit still for a long time?
24. If someone behaves unfairly towards you, do you vigorously defend your interests?
25. Can you slaughter a chicken or a sheep?
26. Does it irritate you if curtains or tablecloths hang unevenly at home, and do you immediately try to straighten them?
27. Were you afraid of being alone at home in childhood?
28. Do your moods often fluctuate without apparent reason?
29. Do you always try to be a sufficiently capable worker in your profession?
30. Do you get angry or lose your temper quickly?
31. Can you be entirely carefree and cheerful?
32. Do you sometimes feel completely happy in a way that seems to permeate you?
33. Do you think you could become a humorous performer (a comedian)?
34. Do you usually express your opinion openly, honestly, and unambiguously to others?
35. Is it difficult for you to tolerate the sight of blood, and does it cause you unpleasant sensations?
36. Do you enjoy work that requires high personal responsibility?

37. Are you inclined to defend those unfairly treated?
38. Do you find it difficult or frightening to go down into a dark basement?
39. Do you prefer work where you have to act quickly but where the quality requirements are not high?
40. Are you sociable?
41. Did you enjoy reciting poetry in school?
42. Did you run away from home as a child?
43. Does life seem difficult to you?
44. After a conflict or offense, have you ever been so upset that going to work seemed simply unbearable?
45. Can it be said that you do not lose your sense of humor when faced with failure?
46. Would you take the first steps toward reconciliation if someone hurt you?
47. Do you love animals very much?
48. Do you check to make sure that you have left your home or workplace in good condition, so nothing happened?
49. Does an unclear thought sometimes haunt you—that something terrible might happen to you or your loved ones?
50. Do you believe that your mood is very changeable?
51. Is it difficult for you to give a report or perform on stage before a large audience?
52. Would you be able to hit someone who insulted you?
53. Do you have a very strong need to communicate with others?
54. Are you the type who falls into deep despair after disappointments?
55. Do you like work that requires energetic organization?
56. Are you persistent in pursuing your goals, especially when facing many obstacles?
57. Can a tragic movie move you to tears?
58. Do you often have difficulty falling asleep because thoughts about daily or future problems keep spinning in your mind?
59. Did you sometimes help classmates or give them answers in school?
60. Would it require a lot of willpower for you to walk alone through a cemetery?
61. Do you carefully keep everything in your apartment in the same place?
62. Does it happen that you wake up in a depressed state after going to bed in a good mood, and this lasts for several hours?
63. Do you adapt easily to new situations?
64. Do you sometimes experience headaches?
65. Do you laugh often?
66. Can you be friendly even toward someone you clearly do not value, do not like, or do not respect?
67. Are you a mobile or active person?
68. Do you worry a lot about injustice?
69. Do you love nature so much that you can consider it a friend?
70. When leaving the house or going to bed, do you check whether the gas is off, the lights are out, and the doors are locked?
71. Are you very fearful?
72. Does your mood change after consuming alcohol?
73. In your youth, did you happily participate in a amateur art circle?
74. Do you see life only pessimistically, without expecting joy?
75. Do you often feel the urge to travel?
76. Can your mood change so sharply that your feeling of happiness suddenly turns into gloom and depression?
77. Is it easy for you to lift the mood of friends in a company?
78. Do you hold a grudge for a long time?
79. Do you mourn others’ misfortunes for a long time?
80. As a school student, did you sometimes rewrite a page in your notebook after accidentally spilling a blot?
81. Do you tend to distrust and be cautious of people rather than trusting them?
82. Do you often have terrifying dreams?
83. Do you sometimes fear that you will be hit by a passing train or, standing at a window of a multi-story building, worry that you might fall out suddenly?
84. Are you usually cheerful in a lively company?
85. Can you distract yourself from difficult problems that need solving?
86. Do you become less reserved and feel more free after drinking alcohol?
87. Are you laconic in conversation?
88. If you had to act on stage, could you immerse yourself in a role to forget that it is only a game?
